# SOX2 is required for inner ear neurogenesis

**DOI:** 10.1038/s41598-017-04315-2

**Published:** 2017-06-22

**Authors:** Aleta R. Steevens, Danielle L. Sookiasian, Jenna C. Glatzer, Amy E. Kiernan

**Affiliations:** 1 Flaum Eye Institute, University of Rochester Medical Center, Rochester, NY USA; 2 Department of Biomedical Genetics, University of Rochester Medical Center, Rochester, NY USA

## Abstract

Neurons of the cochleovestibular ganglion (CVG) transmit hearing and balance information to the brain. During development, a select population of early otic progenitors express NEUROG1, delaminate from the otocyst, and coalesce to form the neurons that innervate all inner ear sensory regions. At present, the selection process that determines which otic progenitors activate NEUROG1 and adopt a neuroblast fate is incompletely understood. The transcription factor SOX2 has been implicated in otic neurogenesis, but its requirement in the specification of the CVG neurons has not been established. Here we tested SOX2’s requirement during inner ear neuronal specification using a conditional deletion paradigm in the mouse. SOX2 deficiency at otocyst stages caused a near-absence of NEUROG1-expressing neuroblasts, increased cell death in the neurosensory epithelium, and significantly reduced the CVG volume. Interestingly, a milder decrease in neurogenesis was observed in heterozygotes, indicating SOX2 levels are important. Moreover, fate-mapping experiments revealed that the timing of SOX2 expression did not parallel the established vestibular-then-auditory sequence. These results demonstrate that SOX2 is required for the initial events in otic neuronal specification including expression of NEUROG1, although fate-mapping results suggest SOX2 may be required as a competence factor rather than a direct initiator of the neural fate.

## Introduction

The vertebrate inner ear is an intricate sensory organ responsible for the perceptions of sound and balance. Critical for transmitting auditory and balance information to higher brain regions is the cochleovestibular ganglion (CVG), consisting of both the spiral (auditory) and vestibular ganglion. In humans, damage or loss of the CVG neurons causes irreversible hearing and balance deficits. Moreover, the success of cochlear implants often depends on the number and relative health of the spiral ganglion neurons^1^. However, despite their clear physiologic importance, it is not well understood how the inner ear neuronal lineage is specified during otic development. CVG neuroblasts are derived from the otic placode, an embryonic structure that gives rise to most of the derivatives of the inner ear, including the sensory hair cells. Beginning at otic cup stages (E9.5 in the mouse) and continuing to late otocyst stages (~E11.5), neuroblasts delaminate from the anteroventral quadrant of the otic cup/otocyst, proliferate, and differentiate into bipolar neurons that innervate both cochlear and vestibular sensory regions^2–5^. The initiation of a neuroblast fate is characterized by a cascade of basic helix-loop-helix (bHLH) proneural gene expression, beginning with neurogenin1 (NEUROG1), closely followed by neurogenic differentiation 1 (NEUROD1). NEUROG1, the neural fate-determining gene, is transiently upregulated in otic precursors and rapidly induces the downstream expression of NEUROD1, a molecule required for neuronal maturation, migration, and survival^6–8^. While it is clear that both these factors are required for neuronal specification and maturation, it is unclear what upstream molecular cues initiate NEUROG1 expression.

The otic neuroblasts derive from the neuro-sensory domain (NSD) in the anteroventral region of the otocyst^2, 9–11^, a region that expresses the High Mobility Group (HMG) transcription factor SOX2^12, 13^. While SOX2 is best known for its role in maintaining stem and progenitor cell pluripotency^14, 15^, it also plays a prominent role in neurogenesis^16–18^. SOX2 is one of the earliest markers of the neural ectoderm and it is highly expressed in neural precursor cells (NPCs), where it supports self-renewal^19^. The importance of SOX2 for both sensory and neuronal development is highlighted in human patients with *SOX2* mutations; these individuals present with a failure of eye formation (anopthalmia), in addition to other neurological symptoms such as hippocampal malformations, severe learning disabilities, epilepsy, and in some cases hearing loss^20–22^.

*Sox2* has been shown to be a critical gene for sensory development in the inner ear^23–26^, although its requirement in the otic neuronal lineage is less clear. Previous studies have shown that overexpression of SOX2 can induce a neuronal phenotype in otic progenitors^25, 27, 28^, and in some studies induce NEUROG1 expression^13^. Interestingly, impairments in the CVG have been observed in some SOX2 loss of function studies^26, 28^, although given the timing of either the deletion or analysis of the otic ganglion, it was difficult to distinguish primary neuronal defects due to loss of SOX2 vs. secondary CVG defects caused by loss of sensory-derived neurotrophic factors. To establish whether there was a primary defect in neurogenesis due to loss of SOX2, we conditionally deleted *Sox2* in the mouse during the period of early inner ear neurogenesis (E8.5-E11.5) and examined the CVG prior to its requirement for sensory derived-neurotrophic factors (E11.75). We found that SOX2 is necessary for the characteristic cascade of proneural gene expression, consisting of NEUROG1 and NEUROD1^7, 8^. Loss of this neuronal gene cascade results in a dramatic reduction of the CVG ganglion by E11.75. Loss of one copy of *Sox2* shows a milder reduction in both proneural gene expression and the size of the CVG, indicating that levels of *Sox2* are critical for the production of the full complement of otic neurons. These results demonstrate that SOX2 is an essential upstream factor during selection of the CVG neurons, although fate-mapping studies demonstrate that SOX2’s temporal expression does not strictly parallel that of NEUROG1, indicating that SOX2 plays a complex role in neuronal specification in the otocyst.

## Results

### SOX2 defines a neurosensory competent region of the otocyst

**Figure 1:**
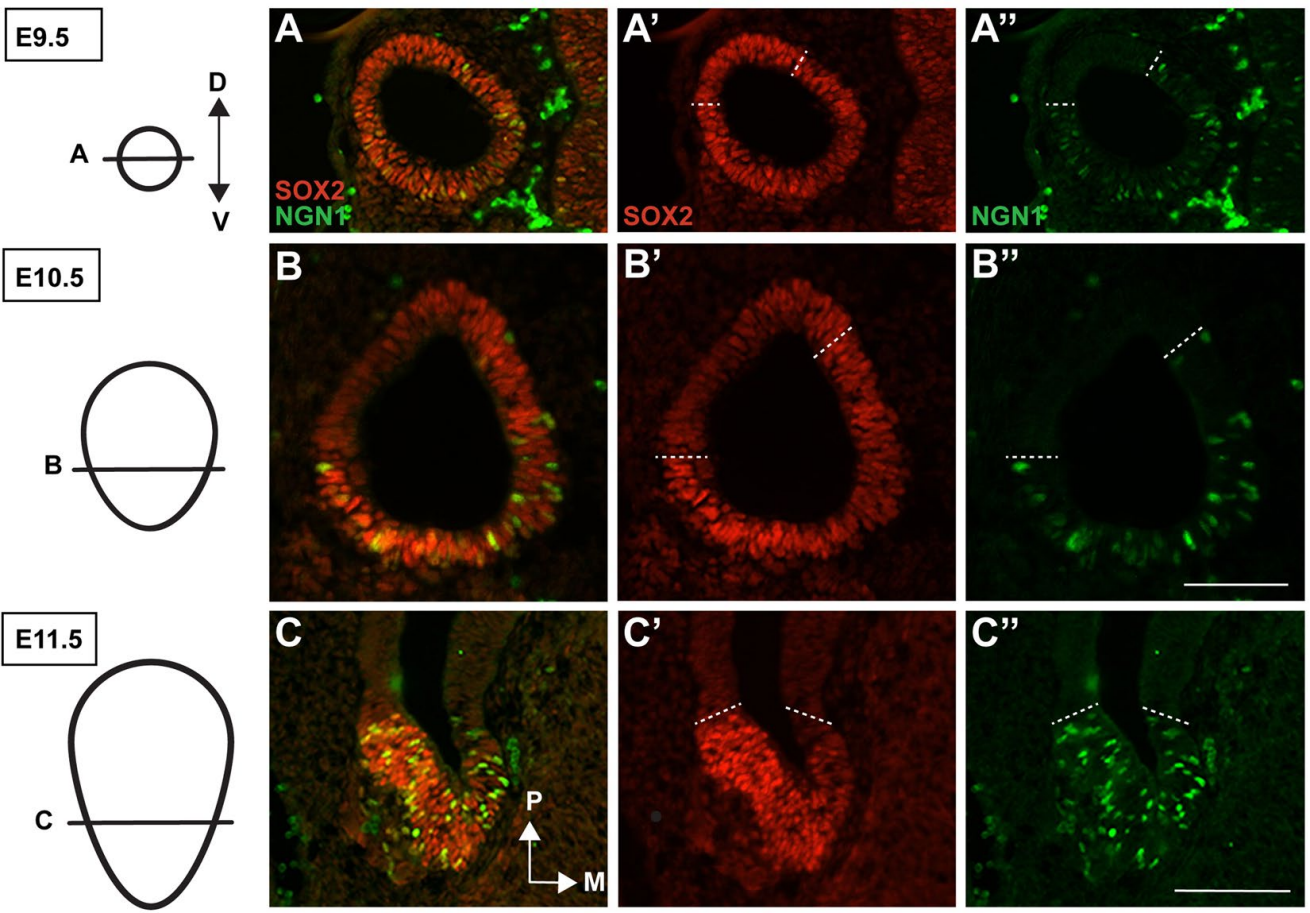
Endogenous SOX2 and NEUROG1 protein show overlapping domains in the mouse otocyst. (**A**–**C**”) Sections through the otocyst of wild type mice, over the developmental timeframe E9.5-E11.5, stained with antibodies to SOX2 and NEUROG1. Note the extensive co-localization of both proteins at all time points. The dotted lines demark the dorsal-most region of overlapping protein expression. Scale bars: 100 μm.

We first examined the extent to which SOX2 expression overlapped with the neurogenic region of the otocyst, defined by NEUROG1 expression^29^. Timed matings were performed, and embryos were harvested for immunohistochemical analysis at E9.5, E10.5, and E11.5, a time series reflective of the period during which the majority of inner ear neurogenesis has been documented to occur^30^ (Fig. 1). As expected from previous reports of SOX2 expression^13, 23, 31^, the SOX2 domain was widespread but generally localized to the more ventral portion of the otocyst at E9.5, where it initially showed a broad anterior-posterior domain. In contrast, NEUROG1 is confined to the anterior half of the otocyst (Fig. 1A,A”) (*n* = 3). A day later at E10.5, SOX2 was downregulated in the lateral region of the otocyst, but remained strong in the anteromedial region, where NEUROG1 is localized (Fig. 1B,B”) (*n* = 3). By E11.5, SOX2 expression is focused in the anteroventral regions, and shows strong overlap with NEUROG1 (Fig. 1C,C”) (*n* = 5). In addition to this region, there is also a SOX2-positive patch in the posterior dorsal region of the otocyst, that does not overlap with NEUROG1 (not shown). This region is likely associated with sensory region generation, which has previously been shown to require SOX2^23^. These results demonstrate that SOX2 and NEUROG1 are overlapping throughout early neurogenesis, although SOX2 initially shows a more broad and diffuse expression pattern. As development proceeds however, SOX2 expression becomes more narrowly focused into two distinct patches: a dorsal posterior region that does not express NEUROG1 (and is therefore likely sensoryrelated) and an anteroventral NSD region that overlaps tightly with NEUROG1.

### SOX2 dose-dependently regulates inner ear neurogenesis

**Figure 2:**
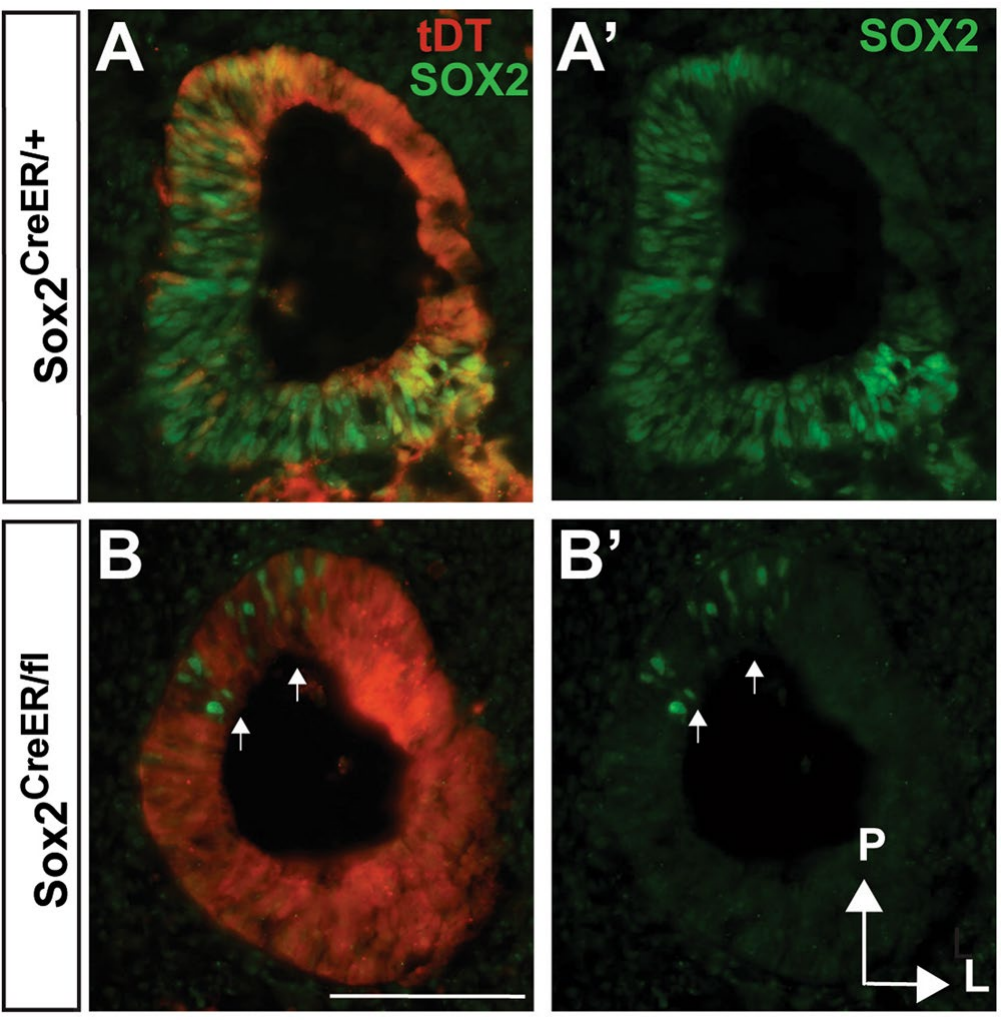
*Sox2* is efficiently deleted in the early otocyst using the *Sox2*-CreER. (**A**,**B**) E11.75 otic sections demonstrating both endogenous SOX2 expression and tdT-reported SOX2 expression from E8.5-E11.5 chronic tamoxifen injections. Note that the majority of the otocyst is labeled by tdT because of SOX2’s more widespread expression at earlier developmental times (E8.5-10.5). In comparison to (**A**’) where widespread SOX2 protein expression is seen in the anterior medial portion of the otocyst (**B**’) shows effective deletion of *Sox2* after chronic tamoxifen administrations in the *Sox2* ^*CreER/fl*^ mutant. Arrows point to the few SOX2-expressing cells that persist in the posterior region of the mutant otocyst that is not in the neurogenic region. Scale bar: 100 μm.

SOX2 coexpression with NEUROG1 within the NSD during early otocyst stages suggests a role for SOX2 in otic neurogenesis, consistent with previous studies^13, 26, 28, 32^. However, the timeframe in which SOX2 is required for otic neurogenesis has not been established. To examine this more closely, we conditionally deleted SOX2 using a tamoxifen-inducible *Sox2-CreERT2* line during the period of otic neuronal specification (E8.5-E11.5)^30^. In addition to deleting SOX2, we included a tdTomato (tdT) reporter line in the breeding scheme so that we could simultaneously fate-map SOX2-expressing cells. To generate Sox2-deleted animals, we crossed a *Sox2-CreERT2* line, in which the SOX2 coding region is replaced by the CreERT2 fusion protein^33^, to a mouse carrying both the *ROSA26-CAGtdT* reporter allele^34^ and the *Sox2* ^*flox*^ allele^35^, thereby generating *Sox2* ^*CreERT2/fl*^ */ROSA26* ^*CAGtdT*^ mice (hereafter these mice will only be identified by their *Sox2* genotype and not by *ROSA26*). Previous studies have shown that the *Sox2-CreERT2* allele recapitulates endogenous SOX2 expression and is a faithful reporter of SOX2 expression^31, 33, 36–38^. Using this breeding paradigm, deletion was generally efficient, with very few SOX2-positive cells remaining 48-hours post-injection (Fig. 2) (*n* = 3). Moreover, the few SOX2-expressing cells that did persist were usually located in the posterior region of the otocyst (Fig. 2B,B’ arrows), a region not associated with neurogenesis.

**Figure 3:**
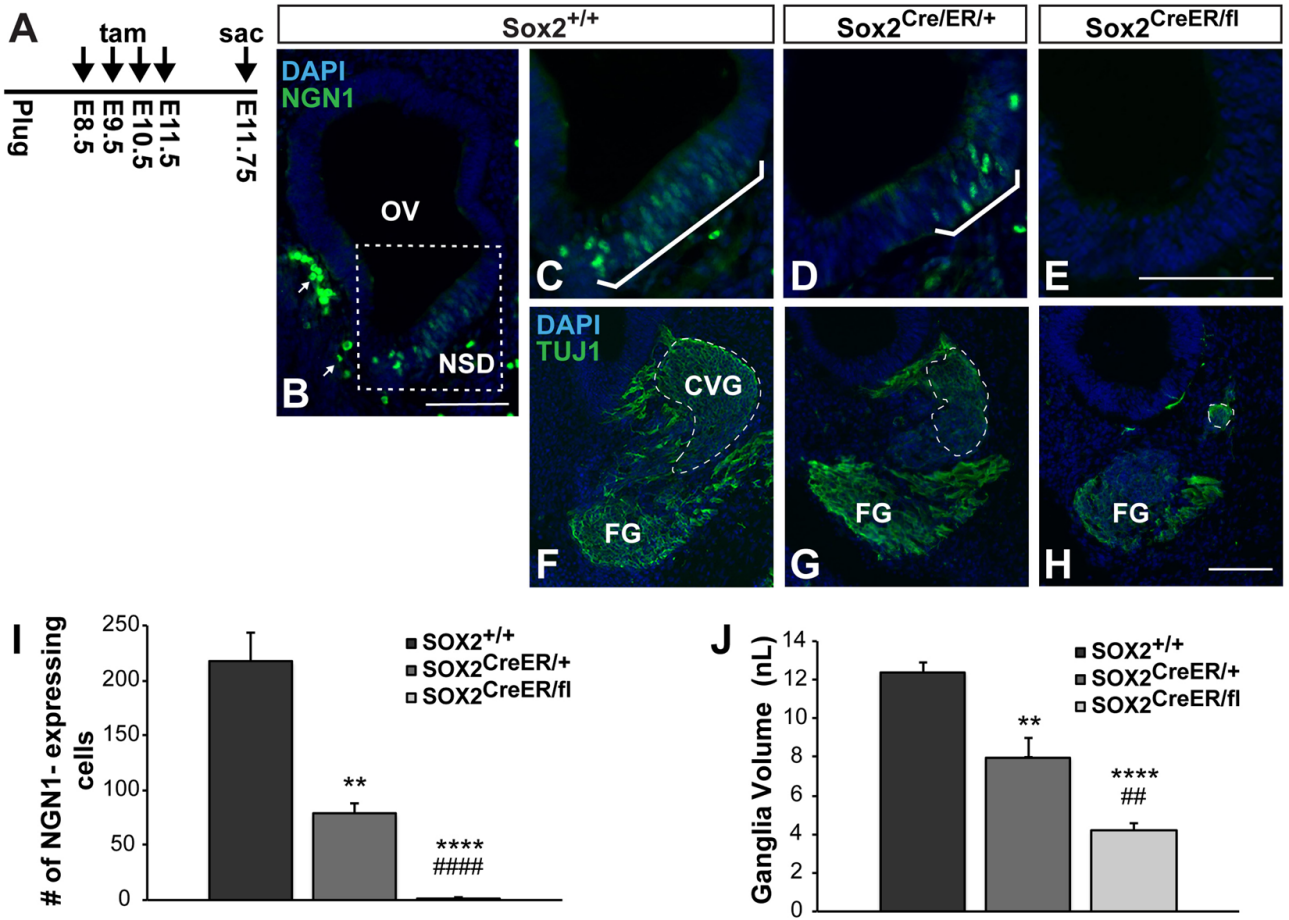
SOX2 dose-dependently regulates inner ear neurogenesis. (**A**) Chronic tamoxifen injection paradigm used for deleting *Sox2* throughout inner ear neurogenesis. (**B**) Low magnification view of the *Sox2* ^+/+^ control otic vesicle, showing the location of the neurosensory domain (dotted box). Arrows point to non-specific staining of blood cells outside the epithelium, which could be easily distinguished from NEUROG1-positive cells based on their cellular shape. (**C**–**E**) Representative sections showing that the number of NEUROG1-expressing neuroblasts (brackets) is reduced according to the *Sox*2 gene dosage. (**E**) The total number of NEUROG1-positive cells was quantified and found to be significantly decreased in both *Sox2* ^*CreER/*+^ mice (***p = 0.0003) (*n* = 7) and *Sox2* ^*CreER/fl*^ mice (****p < 0.0001) (*n* = 7) compared to *Sox2* ^+/+^ controls (*n* = 6), and between *Sox2* ^*CreER/*+^ and *Sox2* ^*CreER/fl*^ mice (^####^ *p* < 0.0001) (one-way ANOVA followed by Student’s *t* test with a Bonferroni correction). (**E-H)** Representative sections showing that the CVG (outlined with dashed lines) is similarly reduced according to *Sox2* genotype. **(I)** The total volume of the CVG was quantified from serial sections and found to be significantly decreased in both *Sox2* ^*CreER/*+^ mice (**p = 0.003) and *Sox2* ^*CreER/fl*^ mice (****p < 0.0001) compared to *Sox2* ^+/+^ controls, and between *Sox2* ^*CreER/*+^ and *Sox2* ^*CreER/fl*^ mice *(* ^##^ *p* = 0.004) (one-way ANOVA followed by a Student’s *t* test with a Bonferroni correction). Error bars represent SEM. OV: Otic vesicle; NSD: Neurosensory domain; CVG: Cochleovestibular ganglion; FG: Facial ganglion. Scale bars: 100 μm.

To delete *Sox2*, tamoxifen was injected daily from E8.5 until E11.5, and embryos were harvested at E11.75, and assessed for markers of neurogenesis (Fig. 3A). In controls, numerous NEUROG1-expressing cells were observed in the anteroventral NSD, as expected (Fig. 3B and C). In contrast, SOX2-deficient inner ears were largely devoid of NEUROG1-positive cells as compared to *Sox2* ^+/+^ controls (Fig. 3B,C,E and quantified in I; *Sox2* ^+/+^ *n* = 6, *Sox2* ^*CreERT2/fl*^ *n* = 7, *p* < 0.0001). Interestingly, the number of NEUROG1-expressing cells was also significantly reduced in *Sox2* ^*CreERT2*/+^ heterozygotes compared to controls (Fig. 3B,C,D and I; *Sox2* ^+/+^ *n* = 6, *Sox2* ^*CreERT2*/+^ *n* = 7, *p* < 0.0001), indicating *Sox2* dosage is important in generating the correct number of otic neurons. These results demonstrate that SOX2 lies upstream of NEUROG1 in otic neurogenesis, and that high levels of SOX2 are required to generate the full complement of CVG neurons.

We next asked whether the reduction in NEUROG1 expression resulted in a smaller or absent CVG in *Sox2*-deleted inner ears. In order to determine the effects of SOX2 loss or reduction on neuronal formation, we labeled the CVG with a marker for early neurons, neuron-specific class III beta-tubulin (TUJ1), and quantified the total volume in serial sections. Through this analysis, we found a similar trend to that observed in the NEUROG1 cell counts, where the total ganglia volume decreased according to the dosage of *Sox2* (Fig. 3F–H and J). We found that the CVG volume was decreased by approximately 70% in *Sox2* ^*CreERT2/fl*^ mutants compared to wildtype controls (Fig. 3J; *Sox2* ^+/+^ *n* = 6, *Sox2* ^*CreERT2/fl*^ *n* = 7, *p* < 0.0001). Moreover, similar to the dose-dependent effects on NEUROG1 expression, we also observed a 30% reduction in CVG volume in the *Sox2* ^*CreERT2/fl*^ heterozygotes compared to controls (Fig. 3F,G and J; *Sox2* ^+/+^ *n* = 6, *Sox2* ^*CreERT2*/+^ *n* = 7, *p* = 0.003). These results demonstrate that the loss of NEUROG1-expressing cells in the *Sox2*-deficient otocyst results in a significant reduction of the CVG ganglion, indicating that SOX2 is required for early specification of the otic neural progenitors.

### Absence of SOX2 impairs downstream expression of NEUROD1

**Figure 4:**
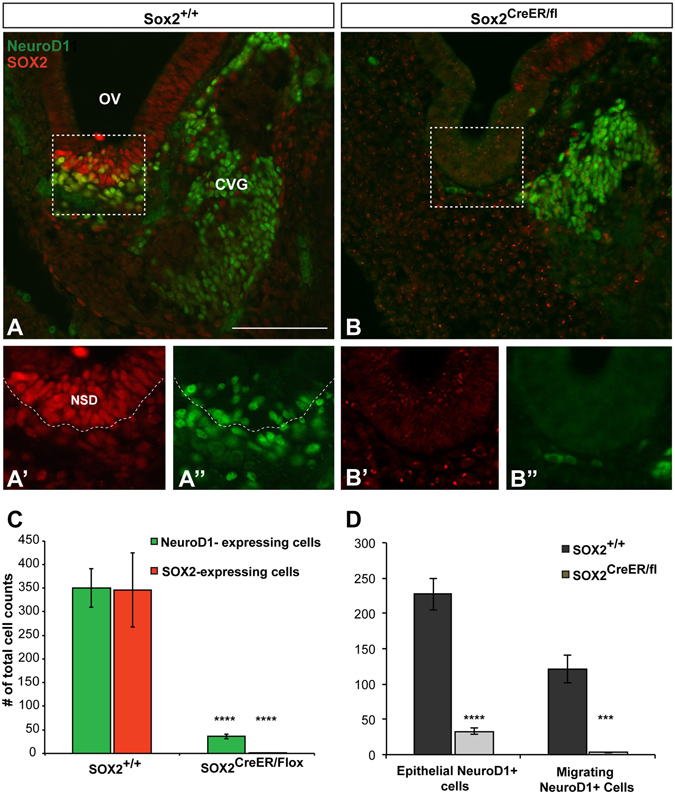
SOX2 is required for the downstream expression of NeuroD1. (**A**) *Sox2* ^+/+^ control section showing the antereoventral portion of the otic vesicle from which neuroblasts delaminate. Boxed region refers to the area in which cell counts were performed. (**A’** and **A**”) show a higher magnification of the neuroblasts contained within the boxed region. Note the extensive colabeling of SOX2 (red) and NEUROD1 (green). Dotted line shows the boundary between the neurosensory epithelium and the mesenchyme. (**B**) Representative section showing the absence of SOX2 and NEUROD1 protein expression in a *Sox2-*deficient mutant. (**B**’ and **B**”) Show a higher magnification of the boxed region from (**B**). Note the absence of immunofluorescence for both SOX2 and NEUROD1. To note, the handful of green cells seen in the mesenchyme did not meet inclusion criteria for neuroblasts and were not counted. (**C**) The total number of NEUROD1 and SOX2-positive neuroblasts was quantified, and both markers were found to be significantly decreased in *Sox2* ^*CreER/fl*^ mice (*n* = 4) compared to *Sox2* ^+/+^ controls (*n* = 3) (****p < 0.0001) (Student’s *t* test). (**D**) Total NEUROD1 cell counts were separated based on either being located in the neurosensory epithelium, or migrating into the mesenchyme; in both cases the number of NEUROD1-positive cells were significantly reduced in the *Sox2* ^*CreER/fl*^ mice (epithelial NEUROD1: ****p < 0.0001 migrating NEUROD1: ***p = 0.0002). Error bars represent SEM. OV: Otic Vesicle; CVG: Cochleovestibular ganglion; FG: Facial ganglion. Scale bars: 100 μm.

In cranial sensory neurons, *Neurog1* has been placed at the top of a hierarchy of a signaling cascade that initiates a sequence of gene expression required for their formation, such that loss of *Neurog1* abolishes expression of all subsequent genes in the pathway^6^. However the dependence of later proneuronal genes on *Neurog1* has not been thoroughly investigated in the ear. To address this, we next asked how loss of *Sox2* during early otocyst stages affects downstream proneuronal genes, by analyzing NEUROD1, which acts as a potent neuralizing agent^39^, in addition to promoting migration^40^ and maturation^6^. Using the previously described experimental paradigm, *Sox2* ^+/+^ control ears and *Sox2* ^*CreERT2/fl*^ *Sox2*-deleted ears were analyzed for expression of NEUROD1 and SOX2. We examined and quantified NEUROD1 expression at E11.75 and found, similar to NEUROG1, a significant reduction of NEUROD1-expressing cells compared to controls (Fig. 4). In *Sox2* ^+/+^ control ears we saw robust expression of NEUROD1 in neuroblasts within the epithelium, as well as NEUROD1-positive migrating neuroblasts detected adjacent to the sensory epithelium, and in the ganglia (Fig. 4A), consistent with NEUROD1’s reported involvement in the multiple stages of neuronal maturation^7, 39, 41^. NEUROD1 and SOX2 were co-localized in almost 100% of the neuroblasts within the epithelium (Fig. 4A,A’,A” and C), supporting previous findings in the chick^13^. Importantly, in *Sox2*-deficient samples, the total number of NEUROD1-positive cells is reduced by approximately 90% (Fig. 4B,B” and C; *Sox2* ^+/+^ *n* = 3, *Sox2* ^*CreERT2/fl*^ *n* = 4, *p* < 0.0001). To determine whether the loss of *Sox2* affected NEUROD1 expression preferentially in the epithelium or during migration, we quantified these areas separately. A severe reduction was observed in both epithelial and migrating neuroblasts in the mutant otocysts (Fig. 4D).

### Neurosensory progenitors die in the absence of SOX2

**Figure 5:**
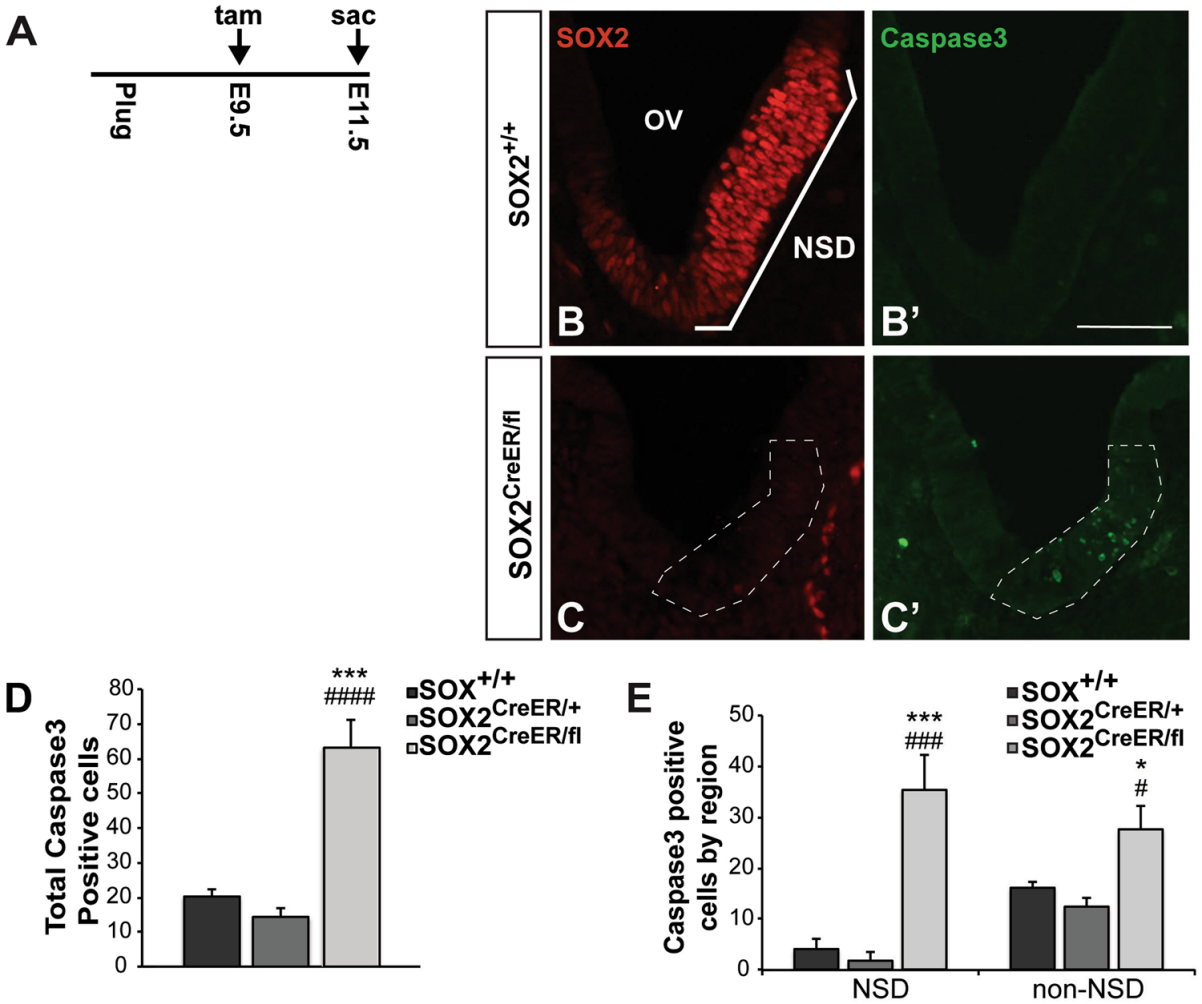
Progenitors die in the absence of SOX2. (**A**) Tamoxifen injection paradigm used for assessing the effect of SOX2 on cell survival. (**B**–**C**’) Representative sections showing SOX2 endogenous protein and activated caspase 3 immunostaining in a *Sox2* ^+/+^ control and *Sox2* ^*CreER/fl*^ mutant. Note the robust increase of caspase 3 in the *Sox*2-deleted mutant (outlined with dashed lines). (**D**) The total number of caspase 3-positive cells was quantified and found to be significantly increased in the *Sox2* ^*CreER/fl*^ mice (*n* = 7) compared to *Sox2* ^+/+^ (*n* = 6) (***p = 0.0005) and *Sox2* ^*CreER/*+^ mice (*n* = 6) (^###^ *p* = 0.0002) (one-way ANOVA followed by a Student’s *t* test with a Bonferroni correction) NSD: Neurosensory domain. Scale bar: 50 μm.

In both the neural retina and hippocampus, SOX2 has been shown to be important for neuronal progenitor survival^19, 42, 43^. In order to determine whether SOX2 is similarly required for otic progenitor survival, we investigated whether there was increased cell death in the *Sox2*-deficient otocysts. For these experiments a single injection was delivered at E9.5, and embryos were harvested at E11.5 and stained for activated caspase 3, a marker of apoptotic cell death, as well as SOX2 in order to assess the extent of *Sox2*-depletion in the mutant (Fig. 5A). Upon analyzing activated caspase 3-stained samples, we found increased labeling in the *Sox2* ^*CreERT2/fl*^ samples, particularly in the NSD region (Fig. 5C and C’). To quantify this increase, total caspase 3-positive cells were counted in all samples, and a significant increase was seen in the *Sox2*-deficient mutant (*n* = 7) compared to controls (Fig. 5D; *Sox2* ^+/+^ *n* = 6, *p* = 0.0005, *Sox2* ^*CreERT2/*+^ *n* = 6, *p* = 0.0002). To ascertain whether the dying cells were likely to be presumptive neuroblasts, total caspase 3-positive counts were divided into posterior dorsal non-neurosensory domains (non-NSD) versus anteroventral neurosensory domains (NSD). This analysis revealed while cell death was mildly increased in the non-NSD, a more dramatic increase was observed in the NSD (Fig. 5E), indicating that a large percentage of the dying cells were likely to be presumptive neuroblasts.

### Sox2 does not recapitulate early NEUROG1 fate mapping

**Figure 6:**
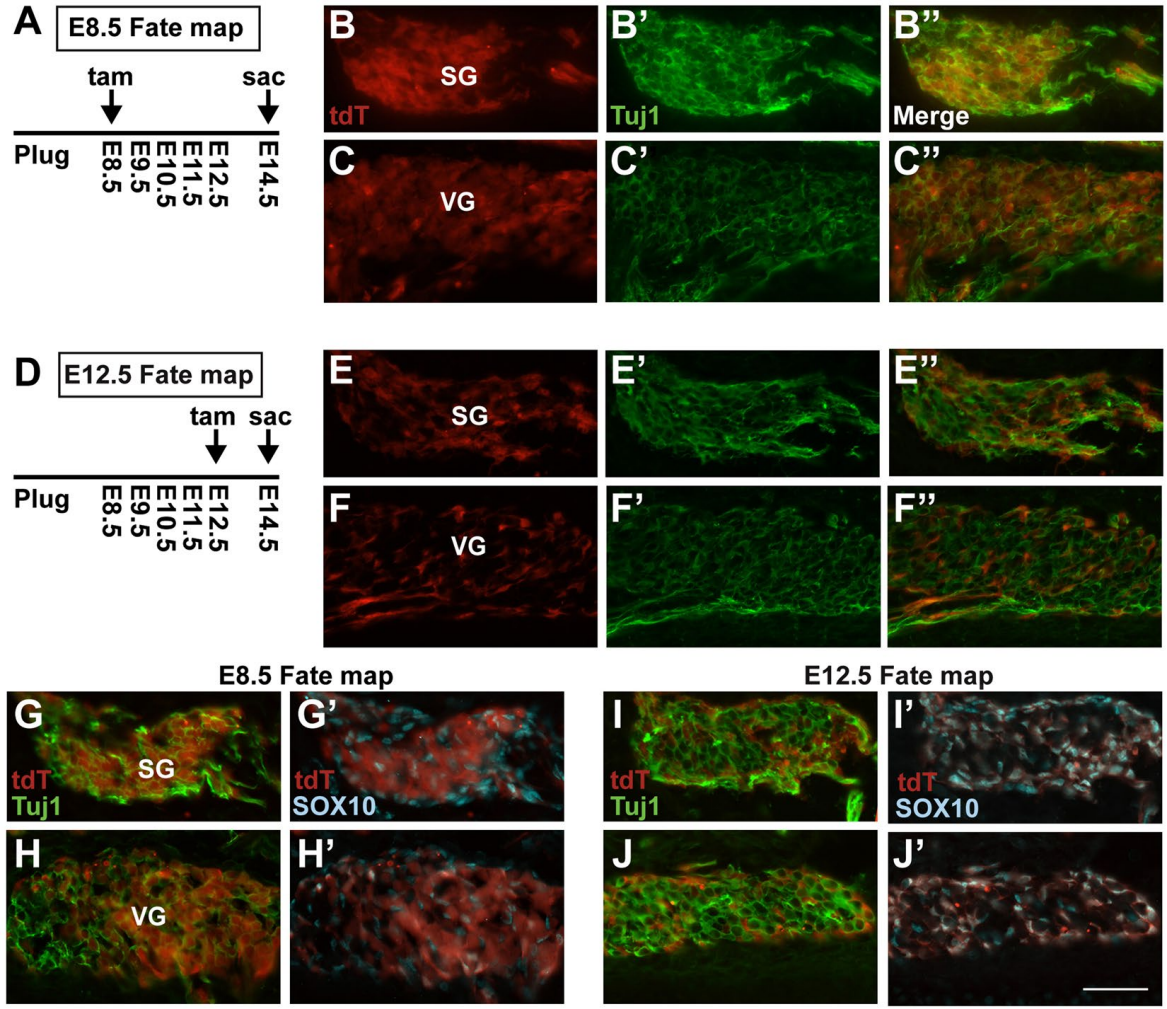
Early (E8.5) SOX2 expression maps to both the cochlea and vestibular ganglia whereas later (E12.5) SOX2 expression maps to the glia. (**A**) Schematic of the early (E8.5) SOX2 fate-mapping timeline. (**B**,**B**”) Representative sections showing E8.5 tdT fate-mapped SOX2 expression in the SG and VG (**C,C”**) at E14.5. Note that tdT reporting specifically labels the neuronal cell bodies in both ganglia. (**D**) Schematic of the later (E12.5) SOX2 fate-mapping timeline. (**E**,**E**”) Representative sections showing E12.5 fate-mapped SOX2 expression reported with tdT in both the SG and (**F,F”**) VG at E14.5. Note that tdT reporting does not overlap with the neuronal cell bodies, but instead appears adjacent to the TUJ1 neuronal marker. (**G**,**H**’) Representative image showing that E8.5 SOX2-reported progenitors contribute to neuronal cell bodies in both the SG and VG at E14.5, and does not overlap with the glia cell marker, SOX10. In contrast (**I**,**J**’) show representative images where E12.5 SOX2 fate-mapped progenitors contribute nearly exclusively to SOX10 labeled glial cells in both the SG and VG. Scale bar: 50 μm.

It is well established during otic development that the vestibular sensory regions develop first, followed by the organ of Corti in the cochlea^5, 44, 45^. A previous fate-mapping study using a NEUROG1-Cre has demonstrated that otic neuronal maturation parallels the sensory maturation, with the cells destined for the vestibular ganglia expressing NEUROG1 initially, followed by cells destined for the spiral ganglion^46^. We wondered whether SOX2 would show a similar temporal pattern of expression in the vestibular ganglia (VG) versus spiral ganglia (SG). By crossing the Sox2-CreERT2 allele with the *ROSA26* ^*CAGtdTomato*^ reporter, we fate-mapped SOX2 expression using Cre-mediated recombination by administering tamoxifen at either E8.5 (when vestibular ganglia begin expressing NEUROG1) or E12.5 (when primarily spiral ganglia express NEUROG1). Embryos were collected at E14.5 (Fig. 6A and D) at which point the ganglia are mature enough to be identified (VG vs SG). Unexpectedly, we found that early (E8.5) SOX2 fate-mapping robustly labeled both the spiral ganglia (Fig. 6B,B”) (*n* = 3) as well as (expectedly) the vestibular ganglia (Fig. 6C,C”) (*n* = 2). This finding is in contrast from NEUROG1 fate-mapping results^46^ and suggests that SOX2 is expressed in spiral ganglia precursors well before NEUROG1 expression is initiated.

We also examined SOX2 reporter expression in the vestibular and spiral ganglia at a later time point, by injecting tamoxifen at E12.5 injection and again harvesting at E14.5. Results of these experiments show many labeled cells in the both the vestibular and spiral ganglion (Fig. 6F) (*n* = 2), although many did not appear to overlap with the neuronal maker TUJ1 (Fig. 6E” and F”). Since previous studies have also reported SOX2 in the neural-crest derived glia in the ganglia^47, 48^, we co-labeled tdT-expressing cells with a glial marker, SOX10^49–51^. Results of this experiment showed that while the majority of cells expressing SOX2 at E8.5 contribute to the CVG neurons (Fig. 6G’,H’), the majority of cells expressing SOX2 at E12.5 become glia (Fig. 6I’J’), as also noted by^31, 52^. Together, these results demonstrate that SOX2 is expressed early throughout both the vestibular and spiral ganglia, in contrast to NEUROG1 fate-mapping results^46^.

## Discussion

SOX2 has been suggested to play a role in promoting inner ear neurogenesis, through both gain and loss-of-function studies, although its exact role in this process has not been clearly elucidated. Our results fill this knowledge gap by demonstrating that SOX2 is necessary during the otic neuroblast specification stage for the formation of the full complement of otic neurons. Specifically, we demonstrate that SOX2 is required for expression of NEUROG1 and NEUROD1, bHLH transcription factors necessary for otic neural development^6–8^. Not surprisingly, given the near-absence of NEUROG1-postive neuroblasts in SOX2-deficient inner ears, the volume of mutant CVG was significantly reduced. Interestingly, our data indicates that SOX2 levels are important for generating the correct number of inner ear neuroblasts. Furthermore, our results place the requirement for SOX2 early in the otic neurogenic signaling cascade, as it is required for the expression of NEUROG1, the earliest marker of the neuroblast fate. However, our SOX2 fate-mapping results do not demonstrate the same temporal pattern of expression in the vestibular and spiral ganglion exhibited by NEUROG1^46^, suggesting that SOX2 may be more of a competence factor than the sole initiator of NEUROG1 expression.

Our results regarding the role of SOX2 in otic neurogenesis are consistent with findings in the brain and other sensory systems, such as the hippocampus and neural retina, that have established a requirement for SOX2 in promoting neural competence^19, 42, 43, 53^. It is currently unclear whether SOX2 directly activates the neural program in the otocyst, or is required as a competence factor. In support of the former, overexpression studies in the chick have found that ectopic expression of SOX2 can expand the NEUROG1-positive domain^13^, and can increase the number of otic neurons^32^. In the mouse, overexpression of SOX2 at later stages in the organ of Corti leads to cells preferentially differentiating as neurons, although these neurons did not express otic-specific neural markers and failed to express later maturation markers^28^. Our fate mapping studies by contrast, indicate that SOX2 is expressed well before NEUROG1, at least in spiral ganglion neuroblasts^46^, suggesting a role as a competence factor. It is possible that the ability of SOX2 to initiate NEUROG1 expression is dependent on protein levels, which fate-mapping does not provide insight into. However, other studies indicate that SOX2 cannot activate the neuronal fate independently. For example, studies by Ahmed *et al*.^27^, demonstrated that ectopic neural differentiation was enhanced when SOX2 was included in combination with the transcription factors SIX1 and EYA1, but was ineffective in inducing neurogenesis on its own. Studies in the lens and embryonic stem cells additionally have shown that SOX2 must partner with specific factors to exert its transcriptional effects^54^. More recent studies in the adult hippocampus have suggested that SOX2 primes the epigenetic landscape by maintaining a bivalent chromatin state and allowing neural differentiation programs to be activated^55^. This type of role has yet to be shown during development, but would be consistent with our results.

Our study found that mice carrying only one copy of *Sox2* produced fewer NEUROG1-positive cells, and impaired neurogenesis in the inner ear. *Sox2* haploinsuffiency has been demonstrated to cause the human condition *SOX2* Anophthalmia Syndrome, a disorder characterized by anophthalmia, learning disabilities, seizures, and postnatal growth failure^22, 56, 57^. In some cases hearing loss has also been reported^22, 58^. Additionally, a number of animal studies have also found that *Sox2* haploinsufficiency produces defects in the eye^43^, the anterior pituitary^58^, and anterior foregut^59^. Two distinct mechanisms of transcriptional regulation by SOX factors have been proposed, including protein-protein interactions and modification of chromatin structure^17^. With regards to SOX2 dependence on protein-protein interactions^54, 60^, it is possible that levels of SOX2 influence which co-factors it partners with, which could produce differences in target gene expression. Alternatively, differences in protein levels may lead to functional consequences by modifying SOX2’s ability to alter DNA confirmation, leading to reduced accessibility for transcriptional complexes. While the molecular mechanism by which *Sox2* dosage regulates cell fate remains to be fully elucidated, our finding of reduced numbers of otic neurons due to *Sox2* haploinsufficiency is consistent with findings in other systems, including human patients with SOX2 mutations, and highlights the importance of protein levels for effective SOX2 function.

Previously, studies have shown that SOX2 is required for the generation of the sensory regions of the inner ear^23, 24^. Here, for the first time, we demonstrate that SOX2 is also required for the specification of the otic neurons. Based on these findings, it is tempting to speculate that SOX2 is required for an early neuro-sensory progenitor, which in its absence, leads to failure of both neural and sensory inner ear components. However, lineage and fate-mapping studies in the ear have not supported the idea of a general neuro-sensory progenitor, although evidence has been shown for a subset of neuro-sensory progenitors that give rise to both neurons and sensory cells in the utricle and saccule^30, 61^. In the absence of evidence for a general neuro-sensory progenitor, it is likely that SOX2 plays specific and distinct roles in both sensory and neural development; however, we currently do not understand the mechanism that determines whether SOX2 specifies neurons or sensory regions. Given that partners of SOX2 can vastly influence which target genes are activated^54^, it is likely that neural vs. sensory may well be dictated by SOX2 co-factors. Factors that have been implicated as interacting with SOX2 include EYA1 and SIX1^27, 62^, both of which are associated with neuronal as well as sensory hair cell formation. Ahmed *et al*.^27^, suggested that interaction with the SWI/SNF chromatin remodeling factors BRG1 and BAF leads to neuronal-specific induction. Another factor that may play a role in determining whether SOX2 acts to specify neural vs. sensory is the Notch signaling pathway. Lateral inhibition has been shown to select a neuronal fate, in which cells that have low Notch activity and high Dll1 express NEUROG1 and become otic neuroblasts^6, 11, 63, 64^. This suggests that Notch-negative and SOX2-positive progenitors will become neurons, whereas Notch-positive and SOX2-positive cells will likely adopt a sensory fate. Consistent with this idea, forced activation of Notch during neurogenesis leads to ectopic hair cells in the otic ganglion^65^, suggesting that Notch activation diverts progenitors from a neuronal fate into a sensory cell fate.

Our results demonstrate that, consistent with other studies, loss of SOX2 leads to cell death^26, 55, 66^. Potentially, SOX2 could function directly as a prosurvival factor. However, it may be more likely that SOX2 is needed to trigger expression of downstream genes necessary for neuroblast specification, and in the absence of the expression of these genes, progenitors undergo cell death. This idea is consistent with studies that showed massive apoptosis in the otic epithelium caused by deletion of either *Neurog1* or *NeuroD1* ^6, 7^, a result the authors hypothesized stemmed from the lack of specification. Similarly, in our findings, given the reduction of NEUROG1 expressing cells in *Sox*2-deleted mutants, it is possible that neural progenitors died from a failure to be specified. Our results are distinct from studies that showed the absence of a ganglia^28^ or neuronal death^26^ in the CVG in more mature *Sox2-*deleted mutants ears. In those studies, *Sox2-*deficient ears were analyzed at later stages, at which point there is also a sensory defect. Thus, the absence/death of neurons at these stages may be attributed to the absence of hair cell targets in the epithelium that provide neurotrophic support rather than a direct reliance on SOX2 for survival. As our study focuses on the role of SOX2 during early otic stages using early specification markers, our results establish a direct requirement for SOX2 in specification of the otic neuroblasts that give rise to the CVG. Further studies will need to be performed to establish whether there is a direct requirement for SOX2 after delamination, although several studies (including ours) have demonstrated an abrupt downregulation of SOX2 in the neuroblasts after delamination until E18/P0^13, 52^.

Our study demonstrates that while SOX2 is required for specifying the vast majority of NEUROG1-expressing neuroblasts, some CVG neurons remain. What then accounts for the remaining neurons? It is possible that the intervals between tamoxifen injections allow enough SOX2 expression to initiate a neuronal program in some cells. However it is also plausible that some neurons develop independently of SOX2 and other factors contribute to the formation of the CVG. Similar to in the neural tube, there could be redundancy between SOXB1 factors^19^. Alternatively, other transcription factors, such as the aforementioned EYA1/SIX1 transcriptional complex, could be responsible for the formation of a portion of the neurons. As discussed, EYA1/SIX1 factors can promote neurogenesis independently of SOX2^27^. Thus, it is possible that a small subset of inner ear neurons can be produced in a SOX2-independent manner.

In conclusion, we have demonstrated that SOX2 is required for the formation of the full complement of CVG neurons, and acts in a dose-dependent manner. These results complement evidence from overexpression studies that have implicated SOX2 in otic neurogenesis^13, 28, 32^. Our fate mapping results show that SOX2 is expressed well in advance of NEUROG1 in the spiral ganglion, raising the possibility that it may be acting as a competence factor rather than a direct regulator. In humans, loss or dysfunction of the spiral ganglion neurons causes permanent hearing impairment, and health of the spiral ganglion is an important feature in cochlear implant success. Thus, deciphering the molecular cascade that leads to inner ear neurogenesis is an important goal for designing future regeneration therapies.

## Methods

All experimental procedures were performed in accordance with guidelines and regulations of the University of Rochester Medical Center. All animal experiments were approved by the University of Rochester’s Committee on Animal Resources.

### Mice and tamoxifen treatment

The following mouse strains were used: *Sox2-Cre* ^*ERT2*^ ^33^, *ROSA26-CAGTdtomato* ^34^, *Sox2* ^*flox*^ ^35^, NEUROG1*-Cre* ^67^. Genotyping was performed by PCR using the following primers: *Sox2-CreER* ^*T2*^ and NEUROG1*-Cre* were both detected with the same primer pair: Cre1F (TGA TGA GGT TCG CAA GAA CC) and Cre1R (CCA TGA GTG AAC GAA CCT GG) yield a 350 bp band. *Rosa26-CAGTdtomato*: tdTomatoF: (CTG TTC CTG TAC GGC ATG G) and tdTomatoR (GGC ATT AAA GCA GCG TAT CC) homozygous mice yield a 196 bp band. *Sox2* ^*flox*^: Sox2Fl WT1 (TGG AAT CAG GCT GCC GAG AAT CC), Sox2Fl WT2 (TCG TTC TGG CAA CAA GTG CTA AAG C), and Sox2FlMut (CTG CCA TAG CCA CTC GAG AAG). Heterozygous mice have both a wildtype 427 bp band and a mutant 546 bp band, whereas homozygous mice have only the 546 bp band. Timed matings were determined by checking for vaginal plugs, and the morning of the plug considered E0.5. Pregnant dams were injected with tamoxifen (3 mg/40 g body weight) and progesterone (2 mg/40 g body weight) at various developmental time points.

### Immunohistochemistry

Embryos were fixed in 4% PFA in PBS buffer at 4 °C for 2.5–4 hours, cryoprotected, and sectioned at a thickness of 16 μm. The sections were incubated with primary antibodies overnight at 4 °C and then incubated with secondary antibodies for 2 hours at room temperature. The primary antibodies used were goat polyclonal α-NEUROG1 (1:700, Santa Cruz), rat monoclonal α-RFP (1:1000, ChromoTek), rabbit polyclonal α-SOX2 (1:500, abcam), mouse monoclonal α-TUJ1 (1:1000, Covance), goat polyclonal α-NEUROD1 (1:1000, Santa Cruz), rabbit α-activated caspase 3 (1:1000, R&D Systems), goat polyclonal α-SOX2 (1:700, Santa Cruz), rabbit polyclonal α-SOX2 (1:500, Millipore), and goat polyclonal SOX10 (1:100, Santa Cruz). The secondary antibodies used were Alexa Fluor 488 donkey α-goat (1:1000), Alexa Fluor 488 donkey α-rabbit (1:1000), Alexa Fluor 555 donkey α-rabbit (1:1000), Alexa Fluor 555 donkey α-rat (1:1000), Alexa Fluor 647 donkey α-mouse (1:1000), Alexa Fluor 647 donkey α-goat (1:1000).

### Quantifications and statistical analysis

*NEUROG1 cell counts and ganglia volume measurements*. NEUROG1-positive cells were counted using Axiovision software (Carl Zeiss). Criterion was set such that cells were counted as NEUROG1-positive only if they had the characteristic teardrop nuclear shape of NEUROG1 and sufficient brightness. The area of the ganglion was measured by tracing the TUJ1 positive ganglia using the outline spline tool in Axiovision software. The total area for each ganglion was converted into a volume by multiplying by the section thickness, 16 μm. *NEUROD1 cell counts*. NEUROD1-positive cells were counted using Axiovision software (Carl Zeiss). A bounded box measuring 100 μm^2^ was placed at the most anterior point of the otocyst and the total number of NEUROD1 + cells in the otic epithelium and surrounding mesenchyme constrained by the bounding box were quantified. NEUROD1 + cells were further categorized by their location (mesenchyme vs epithelium) and co-localization with SOX2.

*Caspase 3 cell counts*. Activated caspase 3 was counted using Axiovision software (Carl Zeiss). Total caspase 3 counts were segregated based on whether the positively labeled cells were located in the anteroventral neurosensory region of the otocyst, that in the controls expressed SOX2 and in the mutant was tdT-positive (termed neurosensory domain (NSD)), versus caspase3 positive cells located in more posterior dorsal regions of the otocyst (termed non-neurosensory domain (non-NSD)).

ANOVA and Student’s *t-*tests were used for statistical analysis. Prism Graphpad 6.0 was used for all statistics.
